# Assessing national vector control micro-planning in Zambia using the 2021 malaria indicator survey

**DOI:** 10.1186/s12936-023-04807-9

**Published:** 2023-11-30

**Authors:** Irene Kyomuhangi, Andrew Andrada, Zhiyuan Mao, Derek Pollard, Christina Riley, Adam Bennett, Busiku Hamainza, Hannah Slater, Justin Millar, John M. Miller, Thomas P. Eisele, Kafula Silumbe

**Affiliations:** 1 School of Public Health and Tropical Medicine, Tulane University, 1440 Canal Street, Suite 2350, New Orleans, LA USA; 2 AKROS, Lusaka, Zambia; 3 PATH, Seattle, WA USA; 4 National Malaria Elimination Centre, Lusaka, Zambia; 5 PATH, Lusaka, Zambia

## Abstract

**Background:**

In 2020, the Zambia National Malaria Elimination Centre targeted the distribution of long-lasting insecticidal nets (LLINs) and indoor-residual spraying (IRS) campaigns based on sub-district micro-planning, where specified geographical areas at the health facility catchment level were assigned to receive either LLINs or IRS. Using data from the 2021 Malaria Indicator Survey (MIS), the objectives of this analysis were to (1) assess how well the micro-planning was followed in distributing LLINs and IRS, (2) investigate factors that contributed to whether households received what was planned, and (3) investigate how overall coverage observed in the 2021 MIS compared to the 2018 MIS conducted prior to micro-planning.

**Methods:**

Households’ receipt of ≥ 1 LLIN, and/or IRS within the past 12 months in the 2021 MIS, was compared against the micro-planning area under which the households fell. GPS points for 3,550 households were overlayed onto digitized micro-planning maps in order to determine what micro-plan the households fell under, and thus whether they received their planned intervention. Mixed-effects regression models were conducted to investigate what factors affected whether these households: (1) received their planned intervention, and (2) received any intervention. Finally, coverage indicators between the 2021 and 2018 MIS were compared.

**Results:**

Overall, 60.0% (95%CI 55.4, 64.4) of households under a micro-plan received their assigned intervention, with significantly higher coverage of the planned intervention in LLIN-assigned areas (75.7% [95%CI 69.5, 80.9]) compared to IRS-assigned areas (49.4% [95%CI: 44.4, 54.4]). Regression analysis indicated that households falling under the IRS micro-plan had significantly reduced odds of receiving their planned intervention (OR: 0.34 [95%CI 0.24, 0.48]), and significantly reduced odds of receiving any intervention (OR: 0.51 [95%CI 0.37, 0.72] ), compared to households under the LLIN micro-plan. Comparison between the 2021 and 2018 MIS indicated a 27% reduction in LLIN coverage nationally in 2021, while IRS coverage was similar. Additionally, between 2018 and 2021, there was a 13% increase in households that received neither intervention.

**Conclusions:**

This analysis shows that although the micro-planning strategy adopted in 2020 worked much better for LLIN-assigned areas compared to IRS-assigned areas, there was reduced overall vector control coverage in 2021 compared to 2018 before micro-planning.

## Background

Malaria remains a significant global public health concern, with an estimated 247 million cases and 619,000 deaths worldwide in 2021. Endemic countries on the African continent account for nearly all of the global burden, with approximately 94% global cases, and 96% of global deaths in 2021 [1]. The persistent high burden of malaria in endemic countries precipitated the “High Burden to High Impact” (HBHI) approach recommended and supported in countries with the highest malaria burden. Importantly, the HBHI approach encourages high burden countries to conduct data-driven sub-national targeting of malaria interventions to bolster impact [2–7].

In Zambia, despite increasing access to diagnosis and treatment of malaria, as well as expansion of vector control efforts [8, 9], there were nearly 9,000 malaria deaths in 2021 [1]. Zambia’s s National Malaria Elimination Programme (NMEP) has deployed several strategies to combat malaria, among which vector control strategies are an essential component. In recent years, vector control in Zambia has primarily consisted of distribution of long-lasting insecticidal mosquito nets (LLINs) and indoor residual spraying (IRS), with small-scale larval source management (LSM) in selected urban and pre-elimination settings. IRS was the primary vector control intervention in the National Malaria Elimination Strategic Plan (NMESP) 2017–2021, while the current NMESP 2022–2026 prioritizes LLINs as the primary vector control intervention [9–11].

In Zambia, LLINs are typically distributed through: mass campaigns; routine distributions through antenatal care (ANC) and expanded programme on immunization (EPI) clinics; and school-based distributions in selected districts, with the vast majority of LLINs are distributed through mass campaigns [12]. IRS is typically conducted in annual campaigns in 115 out of the 116 districts of Zambia, and spraying is usually planned to start in October/November just before the start of the rainy season [10].

Prior to 2020, the strategy for vector control was to achieve universal coverage with LLINs, with IRS deployed in addition to LLINs in selected high-burden areas that were accessible for spraying [10]. In 2020, the NMEP adopted a ‘mosaic’ approach to vector control deployment at the sub-district, health facility catchment level, where some settlements were assigned to receive LLINs during the 2020–2021 mass campaign while others received IRS under a micro-planning strategy [10, 13, 14]. The rationale for this approach was to maximize available IRS and LLIN supplies, and the aim was to ensure households received only one vector control intervention and that co-deployment of the interventions was minimized [13]. Hereafter, the process of assigning areas to receive LLIN or IRS will be referred to as micro-planning, and the designations themselves will be referred to as micro-plans.

Following the shift to a ‘mosaic’ approach in the vector control strategy in 2020, a key interest was in understanding how well the micro-planning was implemented to achieve high vector control coverage with either LLINs or IRS. To answer this primary question, this analysis took data from the 2021 Zambia Malaria Indicator Survey (MIS), and compared what interventions households actually received, against what interventions were planned for their areas based on the NMEP micro-plan maps. The objectives of this study were to: (1) assess how well the micro-planning was followed in distributing LLINs and IRS, (2) investigate factors that contributed to whether or not households received what was planned, and (3) investigate how overall vector control coverage ascertained in the MIS 2021 survey (post-micro-planning) compared to the 2018 MIS survey (pre-micro-planning).

## Methods

The analysis was split into two parts. The first part, which covers objectives 1 and 2, was the micro-planning analysis where coverage indicators from the 2021 MIS were analysed in context of the 2020 micro-planning exercise. The second part, which covers objective 3, compared overall coverage between the 2021 and 2018 MIS without explicitly considering the micro-planning data.

### MIS data and 2020 micro-planning maps

Zambia is divided into ten provinces, which are further divided into 116 districts. For statistical purposes, each district is divided into census supervisory areas (CSAs) and these are in turn subdivided into standard enumeration areas (SEAs) which comprise on average around 23 households. The MIS surveys in Zambia use a 2-stage cluster sampling design where the SEAs serve as ‘clusters’, and are selected at first stage with the probability of selection proportional to cluster population size. The second-stage involves enumerating and then sampling households within selected clusters, where a set number of households in each cluster are randomly selected via simple random sampling for inclusion in the survey [12, 15]. The surveys are conducted in April-May, to coincide with the end of the rainy season and the peak in malaria transmission season. The 2018 MIS included 4177 households across 179 clusters [15], while the 2021 MIS included 4621 households across 202 clusters [12]. A breakdown of the sample sizes for each province is presented in Table 1.

**Table 1:** Sample sizes for households in the micro-planning analysis, and the comparison between the 2021 and 2018 MIS.

	Microplanning analysis with 2021 MIS data		Comparison between the 2021 and 2018 MIS	
	Households included in micro-planning analysis	Households excluded from micro-planning analysis	Total number of households in the 2021 MIS data	Total number of households in the 2018 MIS data
Central	474	1^a^	475	292
Copperbelt	0	467	467	284
Eastern	824	0	824	655
Luapula	461	0	461	666
Lusaka	0	456	456	307
Muchinga	328	0	328	304
North-Western	293	0	293	293
Northern	406	14^a^	420	299
Southern	364	133^a^	497	295
Western	400	0	400	782
**Total**	**3550**	**1071**	**4621**	**4177**

The number of households included (second column) and excluded (third column) from the microplanning analysis are presented. Additionally, the sample sizes for the 2021 and 2018 MIS are presented in the fourth and fifth columns respectively (in bold). ^a^Denotes households that were excluded in the microplanning analysis due to ambiguous micro-planning maps. Details of the sampling design for both the 2021 and 2018 MIS can be found in the respective MIS reports published by the NMEP [12, 15]

The 2020 micro-planning for vector control was carried out at the health facility catchment level, which is the geolocated operational unit for vector control planning and implementation in Zambia. In the 2020 micro-planning exercise, selection of which settlements would receive IRS was based on existing knowledge of structure density, accessibility, suitability of construction material of structures for IRS (including plastered vs. unplastered walls, walls made of reeds/grass, wooden/plank walls), and other operational aspects including feasibility [13]. To ensure each area received at least one of the interventions, IRS/LLIN micro-planning workshops were held in each district. Using spatial data from the Geo-Referenced Infrastructure and Demographic Data for Development (GRID3) program together with population data from the Zambia Statistics Agency (ZamStats), micro-planning maps were developed assigning LLIN or IRS to different health facility catchment areas across the country. Under microplanning in 2020, the NMEP targeted 3.1 million structures for spraying, and 12.9 million people for protection under IRS, while 10.6 million people were targeted to receive LLIN. In this analysis, the micro-planning maps, which were stored in PDF and PNG format, were digitized using QGIS 3.20.3 “Odense” software [16].

**Fig. 1:**
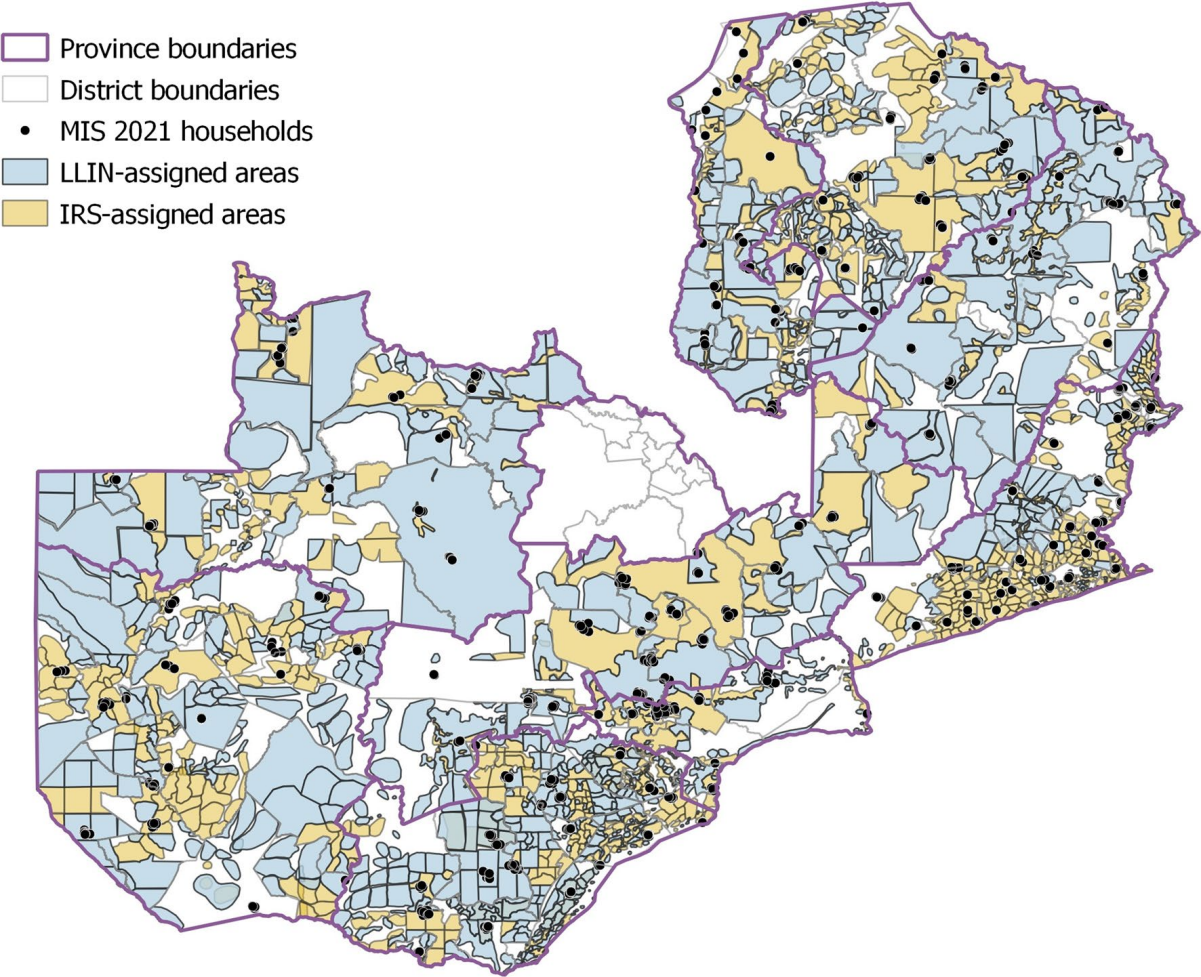
Spatial distribution of 2021 MIS households against the 2020 LLIN and IRS micro-plans. 40.1% [95% CI 32.6–48.0%] of households fell under the LLIN-assigned areas while 59.9% [95% CI 52.0–67.4%] of households fell under IRS – assigned areas

For the micro-planning analysis, GPS points of households in the MIS 2021 data were overlayed onto the digitized maps in order to determine what micro-plan the households fell under (Fig. 1). Households that fell under the LLIN micro-plan and received ≥ 1 LLIN, as well as households that fell under the IRS micro-plan and received IRS within the past 12 months, were deemed to have received their planned intervention.

The micro-planning analysis excluded households from Lusaka province due to limited vector control targeting in this province, households from Copperbelt province were excluded due to missing micro-planning data, and households in clusters with ambiguous micro-planning maps were also excluded from the analysis. These exclusions reduced the total number of households from the MIS 2021 data in the micro-planning analysis from 4621 households to 3550.

### Descriptive statistics for coverage

In this analysis, LLIN coverage was defined as the proportion of households which received ≥ 1 LLIN, while IRS coverage refers to proportion of households that were sprayed in the last 12 months, as per the recommended coverage indicators by the Roll Back Malaria (RBM) Surveillance Monitoring and Evaluation Reference Group. Survey point estimates of coverage were weighted to correct for unequal probabilities of household selection [12, 17], resulting in weights at the cluster level equal to the inverse of the ultimate probability of selection for each household. Empirically estimated standard errors for survey point estimates were estimated using the Taylor linearization method to account for the 2-stage cluster sampling design.

### Regression analysis

Separate mixed effects regression models were conducted to investigate which factors affected whether households under a micro-plan: (1) received their planned intervention, and (2) received any intervention. Therefore, the analysis included two binomial regression models, with a logit link function. In the first model, the outcome variable was binary, indicating whether or not the household received the planned intervention according to the micro-plan in its geography. In the second model, the outcome variable was also binary, indicating whether or not the household received any of the two interventions, regardless of the micro-plan under which it fell.

The covariates in the regression models were selected based on knowledge and experience of intervention deployment in the Zambia context as is presented in the NMEP’s MIS reports [12, 15], as well as availability of data. For example, the micro-planning exercise was conducted at health facility catchment level, therefore travel time to the health facility may have an impact. Lower population density areas are likely to have more rural and harder to reach households, and this may present challenges to receiving the vector control interventions. Additionally, household wealth has historically had an impact on vector coverage indicators [12, 15].

In both models, the fixed effects included: the micro-plan under which the household fell, the average malaria incidence (estimated annual cases per 1000 people from the routine information system) in up to five surrounding health facilities, travel time to the nearest health facility (in hours via walking), population density (people per square kilometre), and household wealth quintiles. Travel time was derived from friction surfaces of travel time across 1 km × 1 km grids from the Malaria Atlas Project (MAP) [18]. Population density was based on the 2020 GRID3 surface, rescaled to sum to approximately 18.4 million (to align with the official Zambian population estimate for 2020) then scaled to 2021 assuming a 3% annual growth rate [19]. The wealth quintiles were derived from raw scores created by assigning a factor weight to a variety of material assets or household characteristics from the MIS data, generated through principal component analysis [12]. Both average malaria incidence and population density were log transformed to improve linearity. Finally, a random effect on cluster was included for both models.

### Comparison between 2021 and 2018 MIS

Coverage indicators were compared between the 2021 and 2018 MIS. Coverage for LLIN was defined as the proportion of households with ≥ 1 LLIN, while for IRS, this was defined as the proportion which received IRS within the past 12 months. This comparison consisted of all 4177 households from the 2018 survey, and the full 4621 households from the 2021 survey.

All statistical analyses were conducted in the R software environment [20]. The regression models were estimated using the *glmer* function in the *lme4* R package [21], while all coverage estimates were obtained and significance tests conducted by a chi-square using *svyby*, *svyciprop* and *svytable* functions in the *survey* package [22].

## Results

### Descriptive statistics for coverage

**Fig. 2:**
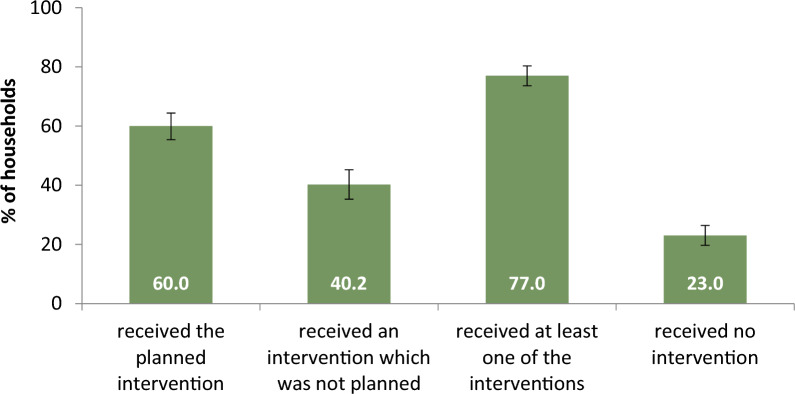
Intervention coverage among 2021 MIS households which were under a micro-plan

Out of the 3,550 households in the micro-planning analysis, a significantly higher proportion fell under the IRS micro-plan (59.9% [95% CI 52.0, 67.4]) compared to the LLIN micro-plan (40.1% [95%CI 32.6, 48.0]). Among all households under a micro-plan, only 60.0% (95%CI 55.4, 64.4) received their assigned intervention; the remaining 40.2% [95%CI 35.3, 45.2] received an intervention that was not planned or no intervention at all (Fig. 2). Among all households, 77.0% [95%CI 73.6, 80.3] received at least LLIN and/or IRS, while the remaining 23.0% [95%CI 19.7, 26.4] did not receive either vector control intervention and had no protection at the time of the 2021 MIS (Fig. 2).

**Fig. 3:**
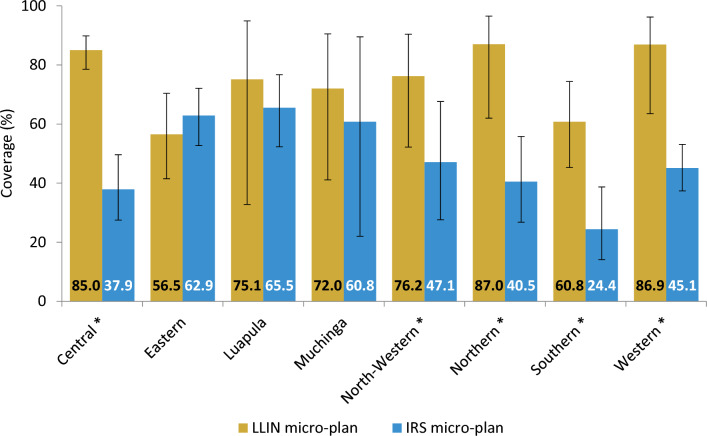
Coverage of planned interventions by province, among 2021 MIS households. LLIN coverage in LLIN-assigned areas was higher than IRS coverage in IRS-assigned areas in Central. * denotes provinces where the difference between LLIN-assigned and IRS-assigned areas is significant with p-value < 0.05 from chi-square analysis. Error bars represent 95% CIs

Households in the LLIN-assigned areas had significantly higher coverage (X^2^ = 244.86, p < 0.001) for their planned intervention (75.7% [95% CI: 69.5, 80.9]) compared to households under the IRS micro-plan (49.4% [95% CI: 44.4, 54.4]). LLIN and IRS coverage varied across provinces, with a general tendency towards higher LLIN coverage compared to IRS coverage. The micro-plan achieved significantly higher LLIN coverage than IRS in 5 of the 8 Provinces included in the analysis: Central (X^2^ = 110.09, p < 0.001), North-Western (X^2^ = 18.92, p = 0.01), Northern (X^2^ = 76.23, p < 0.001), Southern (X^2^ = 47.42, p < 0.001), and Western (X^2^ = 79.25, p < 0.001) provinces (Fig. 3).

### Regression analysis

Regression results show that households falling under the IRS micro-plan had significantly reduced odds of receiving their planned intervention (OR: 0.34 [0.24, 0.48]), and also significantly reduced odds of receiving either intervention (OR: 0.51 [0.37, 0.72]) when compared to households under the LLIN plan (Table 2).

**Table 2:** Odds ratios with associated 95% CIs (within brackets), from the Regression analyses

Covariate	First model: outcome variable is whether or not the household received their planned intervention Odds Ratio (95% CIs)	Second model: outcome variable is whether or not the household received any intervention Odds Ratio (95% CIs)
Micro-plan under which the household fell (reference level is LLIN micro-plan)	0.34 (0.24, 0.48)***	0.51 (0.37, 0.71) ***
Log average malaria incidence	1.05 (0.93, 1.19)	1.15 (1.01, 1.29)*
Travel time to the nearest health facility	1.06 (0.88, 1.28)	1.13 (0.94, 1.36)
Log population density	1.16 (1.05, 1.29)**	1.17 (1.05, 1.30) **
Household wealth quintile	1.21 (1.12, 1.3)***	1.40 (1.30, 1.53)***

Significant codes are as follows: *** indicates p < 0;** indicates p < 0.001; while * indicates p < 0.05

An increase in population density significantly increased the odds of households receiving their planned intervention, or either of the two vector control interventions (OR 1.16 [95%CI 1.05, 1.29] and 1.17 [95%CI 1.05, 1.30], respectively. Meaning an approximate 3 fold increase in the number of people per km^2^ increases the odds of a household receiving their planned intervention by 1.16 times. Similarly, an approximate 3 fold increase in the number of people per km^2^ increases the odds of a household receiving either of the two intervention by 1.17 times. An increase in household wealth quintile also significantly increased the odds of receiving any vector control (OR: 1.21 [95%CI 1.12, 1.3], and 1.40 [1.30, 1.53], respectively) (Table 2).

An increase in average malaria incidence in surrounding health facilities did not have a significant effect on whether or not households received the planned intervention (OR: 1.05 [0.93, 1.19]), but was associated with increased odds of households receiving either intervention (OR: 1.15 (1.01, 1.29)) (Table 2). Meaning that an approximately 3 fold increase in annual malaria cases per 1000 people increased the odds of households receiving either intervention by 1.15 times.

### Comparison between 2018 and 2021 MIS

**Fig. 4:**
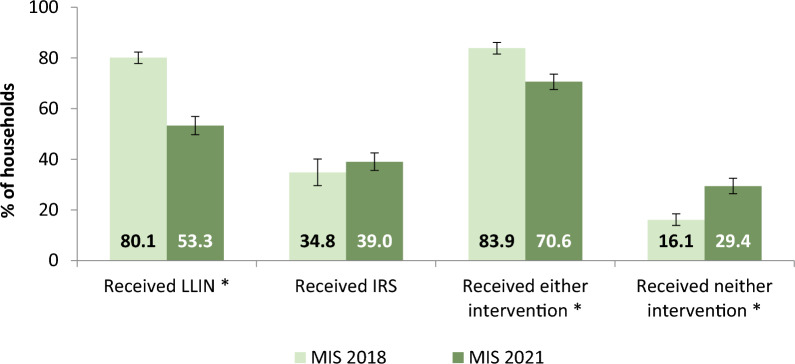
Intervention coverage among all households in the 2018 Vs 2021 MIS. * denotes where the difference in proportion between the 2018 and 2021 surveys is significant with p-value < 0.05 from chi-square analysis. Error bars represent 95% CIs

Comparison between the MIS 2018 and 2021 surveys indicated a decline in overall vector control coverage in 2021. There was a significant decrease in overall LLIN coverage from 80.1% in 2018 to 53.3% in 2021 (X^2^ = 714.21, p < 0.001), as well as a significant decrease in the proportion of households receiving at least one of the vector control interventions (LLIN or IRS) from 83.9% in 2018 to 70.6% in 2021 (X^2^ = 221.52, p < 0.001). Overall, IRS coverage did not change significantly, while the overall proportion of households receiving neither intervention increased significantly from 16.1% in 2018 to 29.4% in 2021 (X^2^ = 221.52, p < 0.001) (Fig. 4).

**Fig. 5:**
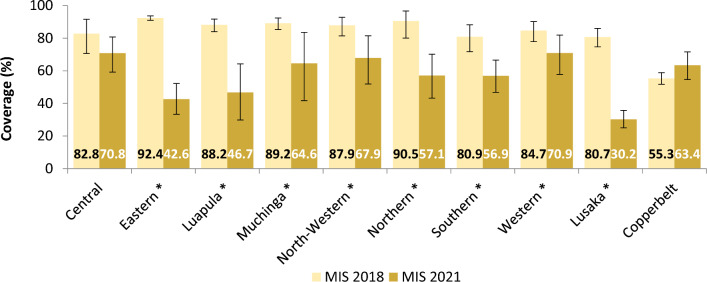
Overall LLIN coverage, by province, in the 2018 Vs 2021 MIS. * denotes where the difference in LLIN coverage between the 2018 and 2021 surveys is significant with p-value < 0.05 from chi-square analysis. Error bars represent 95% CIs

**Fig. 6:**
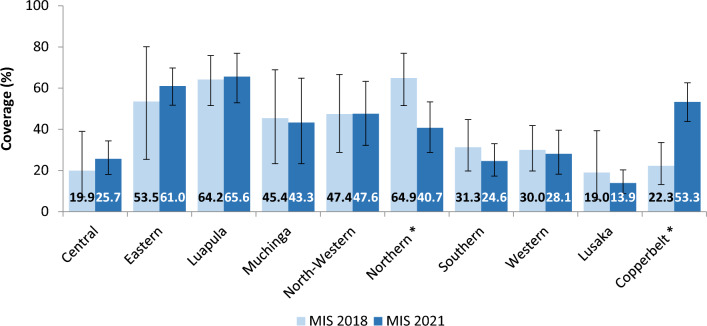
Overall IRS coverage, by province, in the 2018 Vs 2021 MIS. * denotes where the difference in IRS coverage between the 2018 and 2021 surveys is significant with p-value < 0.05 from chi-square analysis. Error bars represent 95% CIs

Breaking down results by province, overall LLIN coverage was higher in the 2018 MIS in 8 of Zambia’s 10 provinces: Eastern (X^2^ = 263.22, p < 0.001), Luapula (X^2^ = 221.33, p < 0.001), Muchinga (X^2^ = 55.76, p < 0.001), North-Western ((X^2^ = 32.31, p = 0.001), Northern (X^2^ = 107.46, p < 0.001), Southern (X^2^ = 53.02, p < 0.001), Western (X^2^ = 53.02, p < 0.001), and Lusaka (X^2^ = 198.14, p < 0.001) provinces, while remaining similar between 2018 and 2021 in Central and Copperbelt (Fig. 5). IRS coverage was similar between the two surveys across all provinces except Northern where there was a significant reduction from 69.4% in 2018 to 40.7% in 2021 (X^2^ = 42.106, p < 0.01) and Copperbelt where there was a significant increase from 22.3% in 2018 to 53.3 in 2021 (X^2^ = 77.22, p < 0.001) (Fig. 6).

**Fig. 7:**
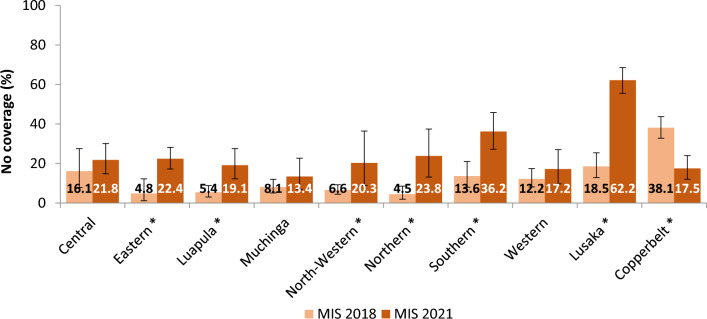
Proportion of households that received neither LLIN nor IRS, in the 2018 Vs 2021 MIS. * denotes where the difference in proportion of households who received neither intervention between the 2018 and 2021 surveys is significant with p-value < 0.05 from chi-square analysis. Error bars represent 95% CIs

Additionally, the proportion of households protected by either an LLIN or IRS decreased significantly between 2018 and 2021 in 6 of 10 Provinces: Eastern (X^2^ = 54.25, p < 0.001), Luapula (X^2^ = 49.21, p < 0.001), North-Western (X^2^ = 22.43, p < 0.01), Northern (X^2^ = 58.37, p < 0.001), Southern (X^2^ = 53.88, p < 0.001), Lusaka (X^2^ = 152.66, p < 0.001. Coverage with either vector control intervention was similar between the survey years in Central, Muchinga and Western provinces, and increased significantly in Copperbelt (X^2^ = 38.30, p < 0.001). The proportion of households without any vector control protection increased significantly between 2018 and 2021 in 6 of the 10 Provinces (Eastern, Luapula, North-Western, Northern, Southern and Lusaka), while remaining similar in Central, Muchinga, and Western provinces, and decreasing significantly in Copperbelt (Fig. 7).

## Discussion

This study sought to investigate how well micro-planning was followed in distributing LLINs and IRS, following the ‘mosaic’ approach to vector control distribution adopted in Zambia in 2020, while also comparing overall vector control coverage between the 2021 and 2018 MIS surveys.

Assessment of coverage indicators for LLINs and IRS showed that under the ‘mosaic’ approach, the LLIN distribution performed significantly better than IRS deployment at achieving high population coverage. This result may be attributable to differences in operational feasibility between LLIN and IRS deployment. The 2020–2021 mass LLIN campaign was concluded just prior to 2021 MIS data collection in April/May, while IRS deployment was scheduled for October/November 2020. IRS deployment has historically had challenges in Zambia. For example between 2017 and 2020, there were operational challenges in IRS deployment leading to delayed spraying in some areas [10] Additionally, IRS coverage has almost always lagged behind LLIN coverage in Zambia [12].

Delayed spraying may have led to spraying efforts falling within the rainy season, which could in turn contribute to low acceptance rates for IRS if households are faced with the inconvenience of packing, arranging and rearranging their household belongings prior to and after spraying [23] during the rains. In Zambia, IRS acceptability in urban areas has also historically contributed to lower IRS coverage when compared to LLIN [10].

Successful IRS deployment is also dependent on the accuracy and efficiency of the enumeration process. The spray teams are required to visit every household in the target area, and when there is no census to guide the teams’ movements, and where implementation area boundaries are unclear, it is likely that households will be missed. Zambia has previously piloted an electronic tool, mSpray (now called REVEAL) [24], which uses satellite imagery for IRS implementation, in select districts in Luapula, Eastern, and Southern provinces with some success [25]. Additionally, IRS campaigns use ‘spray areas’ which are defined by specific numbers or groups of households. Therefore, remote households are likely to be systematically excluded from IRS campaigns.

Another potential operational challenge with the ‘mosaic’ approach is that the micro-planning exercise was conducted at health facility catchment level. Because health facility catchment areas do not have well-defined boundaries that are known by IRS field teams, it is likely to be more operationally challenging than implementation at a more macro-level, such as the district level where clear boundaries are more easily recognized. HBHI countries are encouraged to use sub-national tailoring [26], however if this sub-national tailoring does not use a feasible operational unit, it may not be effective, thus leaving populations in need without essential vector control coverage. A key factor for future consideration is what unit level is operationally feasible for targeting vector control interventions using the ‘mosaic’ approach, while maintaining the advantage of spreading out resources for maximum intervention coverage.

In addition to ensuring households received their planned intervention, a key intention of the micro-planning exercise was to minimize co-deployment of IRS and LLIN. Results indicate that a large proportion of households received an intervention that was not planned (40.2%). This proportion represents households that received the opposite intervention or received an additional intervention to the one they were assigned. Furthermore, 23.1% of households received both vector control interventions. In either case, these proportions represent a substantial deviation from the intention of the micro-planning exercise. It is unclear what factors contributed to these deviations, although it is likely a combination of factors including operational reasons and historical vector control practices on the ground.

Given the lower coverage of IRS compared to LLIN in their respectively assigned areas, it is unsurprising that households falling within IRS-assigned areas had lower odds of receiving their planned intervention. Moreover, results suggest that households within IRS-assigned areas, with the observed challenges to achieving high IRS coverage in these areas, were significantly more likely than LLIN-assigned areas to have no vector control protection, leaving them without any malaria prevention. With prioritization of IRS deployment under the 2020–2021 ‘mosaic’ approach, the risk of households being missed by vector control was higher in the face of underperforming IRS deployment.

Having households missed by vector control coverage is further illustrated in the comparison of coverage between the 2018 and 2021 MIS, where IRS coverage was similar between the two survey years while LLIN declined significantly. Coupled with the decline in overall vector control coverage from the 2018 to the 2021, these results suggest that the focus on IRS coverage under the ‘mosaic’ approach was insufficient to make up for the decline in LLIN coverage. Furthermore, it may be necessary, even in IRS targeted areas, to distribute some LLINs to the most remote households, knowing they will likely be missed by the IRS campaigns.

Increased population density and household wealth quintile had a positive association with households receiving their planned intervention, while they were inversely associated with households not receiving any vector control. This is likely because higher density and wealthier households were closer to health facilities, which operate as operational hubs for LLINs distribution and spaying in Zambia.

Looking at the break-down of results by province, coverage indicators comparing the 2018 and 2021 MIS largely agreed across all provinces except Copperbelt, where instead of a decline in vector control coverage, there was an increase, particularly for IRS. The level of IRS coverage for 2021 was the highest in this province since 2008 [12]. It is not possible to determine whether adherence to the micro-planning was different for Copperbelt compared to the other provinces due to missing micro-planning data for this province. Such information would have greatly added to the interpretation of results.

Finally, while this analysis did not take into account the impact of the COVID-19 pandemic, it is very likely that this event had an impact on the full-scale deployment of interventions leading up to the 2021 MIS, particularly in light of subsequent financial challenges, as well as disruptions to the supply chain for commodities and movement of people caused by the pandemic [10, 12].

Additional iterations of micro-planning and evaluation of the outcomes would be helpful in assessing whether these micro-planning exercises can be done effectively in order to achieve the desired malaria vector control coverage.

## Conclusion

This analysis shows that in the micro-planning strategy used by the Zambia NMEP in 2020–2021 that focused on increasing IRS coverage and reducing the overlap between IRS and LLINs, areas targeted for LLINs achieved significantly higher coverage than those targeted for IRS. Moreover, households in IRS-assigned areas were significantly more likely to have been missed altogether and had unacceptably high levels of being unprotected by any vector control intervention. This suggests that it is more feasible to achieve higher coverage of vector control protection by LLIN mass campaigns as compared to IRS deployment which historically has been challenging to implement to achieve high population coverage. Given that the 2020 micro-planning strategy focused on IRS deployment, these challenges in implementation may have contributed to the poor coverage in IRS-assigned areas, as well as to the decrease in vector control coverage in the 2021 MIS compared to the 2018 MIS.

## Data Availability

Zambia National Malaria Elimination Centre remain the final owners of these data. De-identified data can be requested from the first author by researchers who have been granted permission by the NMEP.
